# Nutritional Management of Patients with Inborn Errors of Metabolism

**DOI:** 10.3390/nu16234154

**Published:** 2024-11-30

**Authors:** María-Luz Couce, Isidro Vitoria

**Affiliations:** 1 Department of Pediatrics, University Clinical Hospital of Santiago de Compostela, Faculty of Medicine, Santiago de Compostela University, 15704 Santiago de Compostela, Spain; 2 IDIS-Health Research Institute of Santiago de Compostela, 15704 Santiago de Compostela, Spain; 3 CIBERER, Instituto Salud Carlos III, 28029 Madrid, Spain; 4 MetabERN, Via Pozzuolo, 330, 33100 Udine, Italy; 5 RICORS-SAMID, ISCIII, 28029 Madrid, Spain; 6 Nutrition and Metabolopathies Unit, La Fe University Hospital, 46025 Valencia, Spain

## 1. Introduction

Inborn errors of metabolism (IEM) are a large group of single-gene disorders resulting from enzyme defects in biochemical and metabolic pathways. While each IMD is rare, they collectively affect  ~1 in 780 births [1,2]. Their main characteristic is their heterogeneity in terms of causes, clinical expression, diagnostic methods, and treatments [3]. Until a few years ago, most of them were lethal or had serious sequelae. Nowadays, although many of them are life-threatening, more and more treatments are becoming available, which are not curative but allow an acceptable quality of life [4].

Nutrition in inborn errors of metabolism (IEM), particularly in intermediary metabolism diseases, is a key element in their treatment to prevent complications, especially neurological ones, and even death. Metabolic imbalance must be corrected and adequate nutritional support provided for normal growth and development, avoiding excessive intake of any toxic nutrient [5,6,7]. All diets prescribed for IEM must be personalized and take into account the patient’s clinical status, tolerances, metabolic stability, age, developmental abilities, and probable prognosis. However, despite advances in recent years, this diet therapy is sometimes very complex, requiring strict control over time that is not always well known. This Special Issue features a total of eleven papers, including two systematic reviews, two narrative reviews, and seven original research articles, which collectively explore the relationship between nutritional status and specific IEM, to identify the best biomarkers for follow-up and personalized treatment.

The systematic reviews reinforce the personalized nutrition strategies in patients with intermediary IEM. One review evaluates the identification genetic variants affecting mineral uptake and metabolism, with a specific focus on iron, to support the implementation of precision nutrition and supplementation strategies [8]. These authors collected 21 studies, leading to an analysis of 22,938 subjects. The results provide comprehensive insights into the association between genetic variants and mineral metabolism, and the findings highlight the relevance of genetic makeup in optimizing health through nutritional interventions. On the other hand, the identification of factors affecting adherence to a low-phenylalanine diet in patients with Phenylketonuria (PKU) was also systematically reviewed [9]. Forty-nine studies were included in the final analysis. The authors conclude that adherence to a low-phenylalanine diet in PKU is a multifaceted behavior influenced by the interplay of family-related, patient-specific, environmental, and therapy-related factors. The impacts of these factors vary across different age groups, emphasizing the need for a personalized, age-appropriate approach to PKU management. Other authors have developed new resources to aid dietary self-monitoring in managing inborn errors of metabolism that require a protein-restricted diet [10].

The narrative reviews are related to nutrition in patients with lipid and amino acid metabolism disorders. The review on the treatment of fatty acid oxidation disorders [11] provides an update of dietary management in basal situations and in stressful situations. The authors emphasize Triheptanoin as a new therapeutic option with a good safety and efficacy profile in long-chain fatty acid oxidation disorders. They report that the use of carnitine remains controversial and new therapeutic options are under investigation. 

The other review is relative to Hyperhomocysteinemia in adult patients as a treatable metabolic condition [12]. It is particularly advisable to determine homocysteine (Hcy) levels for patients who have experienced arterial or venous thrombotic events, as well as those with mild-to-moderate cognitive impairment, neuropsychiatric disorders, or associations with ocular or skeletal abnormalities. A nutritional contribution and supplementation with vitamins B6, B12, folate, and betaine, are vital in order to consistently maintain a plasma Hcy concentration below 50 µmol/L to lower the risk of vascular events.

Among the seven research articles, a key theme involves amino acid metabolism defects. First, a study evaluated the nutrient status and intakes of 34 adults with PKU who had been on diet therapy since infancy, and compared them with control subjects matched for age and sex [13]. The nutrient status and intakes of adult patients with PKU using a protein substitute were adequate for most nutrients (DHA and micronutrients), although lower blood concentrations, compared with controls, were observed for selenium, ubiquinone, and some amino acids. The authors conclude that the regular use of protein substitutes remains a point of attention in adults with PKU, as they are essential to meet DRIs. In a similar line is a study about the long-term efficacy and use of phenylalanine-free infant amino acid formula [14]. All specialist infant formula should be systematically evaluated to ensure its safety, tolerance, and acceptance. In this study, it is shown that the prescription of PKU Start: formula (PFIF) should be mainly replaced with a second-stage weaning protein substitute before 2 years of age to optimize PKU dietary management. In addition, two articles show the applicability and potential of breastfeeding and inborn errors of amino acid and protein metabolism. One article describes a tool that enables the calculation of the amount of human milk and special formula required for infants aged 0–6 months with intermediary IEM [15]. By entering the infant’s body weight and the requirements for the limiting amino acid or intact protein, the tool provides an estimate of the required human milk volume. The values generated by the spreadsheet serve as general guidance only and must be individualized for each patient, considering the severity of their disorder. The authors note that in cases of severe enzyme deficiency, caution should be exercised when interpreting the spreadsheet results, and adjustments should be made based on the specific biochemical parameters associated with each IEM. In this line, Kowalik et al. [16] indicate that maintaining lactation and extending the period of feeding the infant with human milk in the first 6 months of life is possible by breastfeeding on demand, under regular biochemical monitoring: preferably weekly in PKU infants, and at least every 2–4 weeks in infants with other IEM. These two articles recommend that breastfeeding be encouraged more frequently, as there is mounting evidence to suggest that breastfeeding is a safe practice for infants with amino acid IEM. In another study, Boyle et al. [17] provide useful data on the protein and amino acid content of fruits, vegetables, and starchy roots which can be used in clinical practice. In this context, it is known that fruits and vegetables are crucial for health due to their high content of essential vitamins, minerals, and antioxidants and their low protein and amino acid content, and they are therefore considered beneficial in the dietary management of inborn errors of amino acid and protein metabolism.

Recent studies on propionate defects (PDs) have also explored innovative approaches [18], which suggest that PD patients be managed as immunocompromised independently of their nutritional status or metabolic state (compensated or decompensated). There is a significant gap in the understanding of the humoral immune response of PD patients, and the aim of this study was to explore the immunological function of these individuals. Hypogammaglobulinemia G is observed in these patients. The authors propose the incorporation of immunoglobulin quantification in the current guidelines for diagnosing and managing PD patients and highlight the need to consider them as immunocompromised individuals who need prophylactic measures such as gamma globulin and antibiotic administration. Finally, a study about anthropometric, body composition, and nutritional indicators with and without nutritional intervention during nitisinone therapy in alkaptonuria [19] shows that ongoing dietetic intervention prevented the loss of lean body mass despite protein restriction and moderated serum tyrosine increase, leading to less prevalent corneal keratopathy.

## 2. Conclusions

This compilation of articles has significantly expanded the depth of our understanding of nutrition as a basic tool for patients with intermediary IEM. The prominent role of nutrition, in particular, personalized nutrition, in this Special Issue demonstrates the importance of avoiding excessive intake of any toxic nutrient with implications for normal growth and development. Nevertheless, clinical studies with a longer follow-up are needed to establish recommendations to provide an adequate, balanced nutritional supply of energy, proteins, vitamins, and other nutrients, and thus to improve growth and development and short- and long-term outcomes.

## References

[1] Sanderson S., Green A., Preece M.A., Burton H. (2006). The incidence of inherited metabolic disorders in the West Midlands. UK Arch. Dis. Child..

[2] Tiivoja E., Reinson K., Muru K., Rähn K., Muhu K., Mauring L., Kahre T., Pajusalu S., Õunap K. (2022). The prevalence of inherited metabolic disorders in Estonian population over 30 years: A signifcant increase during study period. JIMD Rep..

[3] Vergano S.A.S. (2022). Inborn Errors of Metabolism: Becoming Ready for Rare. Pediatr. Rev..

[4] Saudubray J.M., Garcia-Cazorla À. (2018). Inborn Errors of Metabolism Overview: Pathophysiology, Manifestations, Evaluation, and Management. Pediatr. Clin. N. Am..

[5] Evans M., Truby H., Boneh A. (2017). The relationship between dietary intake, growth and body composition in Phenylketonuria. Mol. Genet. Metab..

[6] Balci M.C., Karaca M., Yesil A., Selamioglu A., Korbeyli H.K., Durmus A., Ak B., Kozanoglu T., Hacioglu I., Gokcay G.F. (2023). Evaluation of the risk factors for noncommunicable diseases in patients with inborn errors of amino acid metabolism receiving nutrition therapy. J. Pediatr. Endocrinol. Metab..

[7] Maier E.M., Dokoupil K. (2022). 3.19 Inborn Errors of Metabolism. World Rev. Nutr. Diet..

[8] Bösch E.S., Spörri J., Scherr J. (2024). Genetic Variants Affecting Iron Metabolism in Healthy Adults: A Systematic Review to Support Personalized Nutrition Strategies. Nutrients.

[9] Yagudina R., Kulikov A., Serpik V., Protsenko M., Kopeyka K. (2024). Factors Affecting Adherence to a Low Phenylalanine Diet in Patients with Phenylketonuria: A Systematic Review. Nutrients.

[10] Franceschetto B.F., Orlandi J.M., Rosa W.C., Scortegagna M.L., Farret L., Schwartz I.V.D., Poloni S. (2024). AminoApp: The First Brazilian Application for Dietary Monitoring of Inborn Errors of Metabolism in Patients on a Low-Protein Diet. Healthc. Inform. Res..

[11] Peña-Quintana L., Correcher-Medina P. (2024). Nutritional Management of Patients with Fatty Acid Oxidation Disorders. Nutrients.

[12] González-Lamuño D., Arrieta-Blanco F.J., Fuentes E.D., Forga-Visa M.T., Morales-Conejo M., Peña-Quintana L., Vitoria-Miñana I. (2023). Hyperhomocysteinemia in Adult Patients: A Treatable Metabolic Condition. Nutrients.

[13] Venegas E., Langeveld S., Ahring K., Benitez R., Desloovere A., Dios E., Gómez E., Hermida A., Marsaux C., Verloo P. (2024). Nutrient Status and Intakes of Adults with Phenylketonuria. Nutrients.

[14] Yilmaz Nas O., Ashmore C., Evans S., Pinto A., Daly A., Yabancı Ayhan N., MacDonald A. (2024). Phenylalanine-Free Infant Formula in Patients with Phenylketonuria: A Retrospective Study. Nutrients.

[15] Vitoria-Miñana I., Couce M.L., González-Lamuño D., García-Peris M., Correcher-Medina P. (2023). Breastfeeding and Inborn Errors of Amino Acid and Protein Metabolism: A Spreadsheet to Calculate Optimal Intake of Human Milk and Disease-Specific Formulas. Nutrients.

[16] Kowalik A., Gudej-Rosa S., Nogalska M., Myszkowska-Ryciak J., Sykut-Cegielska J. (2024). Breastfeeding in PKU and Other Amino Acid Metabolism Disorders-A Single Centre Experience. Nutrients.

[17] Boyle F., Lynch G., Reynolds C.M., Green A., Parr G., Howard C., Knerr I., Rice J. (2024). Determination of the Protein and Amino Acid Content of Fruit, Vegetables and Starchy Roots for Use in Inherited Metabolic Disorders. Nutrients.

[18] López-Mejía L.A., Vela-Amieva M., Guillén-López S., Mancera-Hernández D., Ibarra-González I., Medina-Torres E.A., Espinosa-Padilla S.E., Fernández-Lainez C. (2024). Hypogammaglobulinemia Class G Is Present in Compensated and Decompensated Patients with Propionate Defects, Independent of Their Nutritional Status. Nutrients.

[19] Ranganath L.R., Khedr M., Milan A.M., Davison A.S., Hughes A.T., Norman B.P., Bygott H., Luangrath E., Judd S., Soulsby C. (2024). Anthropometric, Body Composition, and Nutritional Indicators with and without Nutritional Intervention during Nitisinone Therapy in Alkaptonuria. Nutrients.

